# Trial Readiness: Understanding the Natural History of Rare Diseases

**DOI:** 10.1002/jimd.70102

**Published:** 2025-10-23

**Authors:** Thomas Opladen, Ulrike Mütze, Florian Gleich, Sven F. Garbade, Oya Kuseyri Hübschmann, Matthias Zielonka, Stefan Kölker

**Affiliations:** ^1^ Center for Pediatric and Adolescent Medicine Department I, Division of Pediatric Neurology and Metabolic Medicine Heidelberg University, Medical Faculty Heidelberg, and Heidelberg University Hospital Heidelberg Germany

## Abstract

Inherited metabolic diseases (IMD) represent the largest and still growing group of treatable genetic disorders and are increasingly amenable to targeted interventions that achieve varying degrees of prognostic improvement. Innovative therapies are on the horizon and offer promising opportunities for disease‐changing treatment for a variety of IMDs. For the development of clinical trials specifically for IMDs and in the context of trial readiness, a thorough understanding of the natural history of the IMDs is indispensable for an objective evaluation of meaningful improvement of novel treatment options. Patient registries are key instruments in this regard, since they are recognized as powerful instruments for the collection of longitudinal real‐world data, elucidating the phenotypic diversity of disease courses, understanding the impact of diagnosis and treatment on clinical outcomes, and investigating prognostic factors. At the same time, they enable the collection of patient‐specific outcome parameters (PROMs) that improve the understanding of the natural phenotype in rare diseases by identifying clinically relevant endpoints, disease burden over time, unmet medical needs, and the impact of diseases and prescribed diets and medication on the quality of life of patients and caregivers. Meta‐analysis and quantitative retrospective natural history modeling allow the evaluation of the disease course with the help of published aggregate data, where patient registries are not available. Finally, the various data sources provide the theoretical basis for practical applications such as the creation of consensus‐based guidelines, pass studies, and mathematical modeling. This review describes the various options for evaluating and understanding the natural history of rare IMDs in detail, with the ultimate aim of achieving adequate trial readiness.

**Abbreviations**
AI: artificial intelligence
ASA: argininosuccinic aciduria
ATMP: advanced therapy medicinal products
CBS: cystathionine β‐synthase
CDE: common data elements
CLN: ceroid lipofuscinosis
CRS: Clinical Rating Scale
CSF: cerebrospinal fluid
CTLN1: citrullinemia type
E‐HOD: European Network and Registry for Homocystinuria and Methylation Defects
E‐IMD: European Registry and Network for Metabolic Intoxication Diseases
EMA: European Medicines Agency
ERDRI: European Rare Disease Registry Infrastructure
ERT: enzyme replacement therapies
FAIR: findable, accessible, interoperable, reusable
GDPR: General Data Protection Regulation
HCO: healthcare providers
HMDB: Human Metabolome Database
HPO: Human Phenotype Ontology
HRQOL: health‐related quality of life
IMD: inherited metabolic diseases
iNTD: International Working Group on Neurotransmitter‐related Disorders
MAH: marketing authorization holders
MetaERN: European Reference Network for Hereditary Metabolic Disorders
ML: machine learning
NKH: nonketotic hyperglycinemia
OTC: ornithin transcarbamylase
PASS: postauthorization safety studies
PRAC: Pharmacovigilance Risk Assessment Committee
PREMS: patient‐reported experience measures
PROMS: patient‐reported outcomes measures
QUARNAM: quantitative retrospective natural history modelling
RWE: real‐world evidence
SAT: single arm trial
SMA: spinal muscular atrophy
SoC: standard of care
UBDRS: Unified Batten Disease Rating Scale
UCD: urea cycle disorder
U‐IMD: Unified Registry for Inherited Metabolic Disorders
WMB: whole body model

## 1 Introduction

Inherited metabolic diseases (IMDs) are a heterogeneous group of disorders caused by inherited deficiency of proteins and organelles involved in the synthesis, breakdown, and transport of specific metabolites. According to the Inborn Errors of Metabolism Knowledgebase (IEMbase, http://www.iembase.org), the most comprehensive online knowledge base for IMDs, close to 2000 IMDs (as of May 2025) have been identified to date [1]. With modern analytical high throughput technologies, the molecular and metabolic basis of hitherto etiologically unknown diseases has been solved and, therefore, the number of IMDs is still increasing [2]. At the same time, IMDs represent the largest group of treatable genetic disorders and are increasingly amenable to targeted interventions [3, 4], offering varying degrees of prognostic improvement. Established treatment strategies include balanced dietary treatment that aims to limit the intake of precursors of endogenous toxins and to substitute missing compounds of synthetic pathways. The supplementation with cofactors (e.g., pyridoxin [vitamin B_6_] or tetrahydrobiopterin [BH_4_]) aims to enhance residual enzyme activity. Orphan drugs open alternative pathways for the detoxification of toxic compounds, such as nitrogen scavengers for the detoxification of ammonium and betaine for lowering homocysteine concentrations, provide substrate reduction for complex molecules such as miglustat for Gaucher disease [5] or hepatotoxic metabolites such as nitisione for tyrosinemia type 1. Enzyme replacement therapies (ERT) have opened new therapeutic avenues for individuals with lysosomal storage disorders since the 1990s by repetitive provision of a functional version of the defective enzyme to restore the respective metabolic pathway [6]. A more sustained but also more invasive form of enzyme replacement is solid organ transplantation, such as liver transplantation in urea cycle disorders [7] or hematopoietic stem cell therapy in mucopolysaccharidosis type 1. Although many of the above‐mentioned therapeutic strategies are disease‐changing and have a significant long‐term benefit for affected individuals with IMDs, the application of analogous strategies for other diseases of the same disease group may fail to be similarly beneficial; although reaching certain biochemical and clinical endpoints in clinical trials, not all of them being meaningful for affected individuals [8, 9]. Sometimes therapeutic strategies may fail to reach their goal as they restrict flexibility and are difficult to adhere to on a day‐to‐day basis, such as dietary treatment. As a consequence, years and decades after their launch, it has become obvious for most available therapeutic strategies for IMDs that although attenuating the clinical phenotype at variable degrees, they cannot reliably protect against irreversible organ damage and impaired quality of life.

Innovative therapies, such as gene replacement therapy, mRNA therapy, and antisense oligonucleotide therapy, might be able to overcome these current limitations. Advanced therapy medicinal products (ATMPs) defined as medicines for human use that are based on genes, tissues, or cells, constitute a group of novel therapeutic techniques [10]. ATMPs can be subdivided into (a) gene therapy medicines, (b) somatic cell therapy medicines, and (c) tissue‐engineered medicines. In recent years, ATMPs have transformed the management of previously incurable diseases. They offer causal treatment, disease modification, and reduction of mortality and long‐term morbidity [11, 12]. Available gene therapies already cover a broad spectrum of diseases and indications. Further approvals of products in advanced stages of clinical trials are expected in the near future.

However, in the real world there are snags, particularly in the area of rare IMDs. Developing clinical trials specifically for IMDs and objectively evaluating the efficacy of innovative therapies for IMDs is challenging due to various factors: (1) IMDs are generally rare and hence patients are low in number and often geographically scattered, challenging the feasibility of clinical trials. (2) Individuals with IMDs often present with a broad clinical spectrum, sometimes even in individuals with the same pathogenic gene variants [13]. This increases the risk of making incorrect assumption about the true effect size of trial drugs. For instance, the therapeutic benefits may be over‐estimated in screened individuals with a naturally occurring attenuated phenotype who are presymptomatically identified and prospectively treated. (3) The understanding of long‐term disease trajectories is incomplete. Furthermore, prognostic biomarkers that enable to reliably predict disease progression at early stages of the disease and individually adjust therapies during lifetime are unknown for the majority of IMDs [14, 15, 16]. (4) Finally, apart from the controversially discussed medication costs [17], there is still uncertainty about the long‐term benefits and safety of innovative therapies, and cost models are rarely based on true costs and scarcely include the dimension of societal costs.

Longitudinal observational studies using industry‐independent patient registries, which are semantically interoperable and follow the FAIR principles (findable, accessible, interoperable, reusable; [18]), are being conducted by international scientific networks, involve patient organizations, and are powerful tools to overcome some of these limitations. They are considered the currently best way to collect comprehensive real‐world data and achieve sufficient sample sizes for epidemiological and clinical research for rare diseases in general and IMDs in particular [14]. Besides precise phenotyping and the evaluation of current diagnostic and therapeutic strategies, data from patient registries can also be used for individual prediction of disease severity and progression, integrating biochemical data and other data sources. Unfortunately, only a subset of existing registries currently meets minimum quality criteria [19] and enables data exchange in a GDPR‐compliant way, hampering the combination of existing small cohorts to build cohorts with sufficient sample size. Furthermore, there is a need to define core outcome sets to publish results of clinical studies in a structured way [20], facilitating systematic literature search, meta‐analysis [21, 22], and recursive natural history modelling [23].

This overview elucidates strategies for evaluating and understanding the natural course of rare IMDs with the aim of achieving adequate study maturity. It emphasizes that study readiness in the field of IMDs requires tailored approaches to study design and detailed assessment of the natural history. In the latter, consideration of the patient's perspective is becoming increasingly important. Practical aspects such as patient selection and recruitment, predictive models and scoring systems for assessing disease severity as well as quality‐assured follow‐up and postauthorization studies are highlighted.

## 2 Real World Data: Patient Registries for Understanding the Natural History of Rare Diseases

Patient registries are recognized as powerful tools for assessing the natural course of a disease, understanding differences in diagnosis, treatment, and outcomes, and investigating factors that affect prognosis and quality of life. They are therefore indispensable for understanding the natural history of rare diseases and thus for clinical readiness [14].

Initial systematic collections of patient data were limited to observational studies at single centers or single diseases. Furthermore, many registries were industry‐led drug registries (sometimes with multiple registries for a single disease) instead of industry‐independent disease registries. This ultimately led to data fragmentation and data duplication, with the result that these studies often failed to achieve the critical mass needed to elucidate phenotypic diversity and decipher the efficacy and safety of diagnostic and therapeutic strategies. This changed in the context of the increased political attention paid to the importance of disease registries and financial support through European funding programs. Among others, national and international patient registries for IMDs such as the European Registry and Network for Metabolic Intoxication Diseases (E‐IMD: https://www.eimd‐registry.org), the European Network and Registry for Homocystinuria and Methylation Defects (E‐HOD: https://www.ehod‐registry.org), the Registry of the International Working Group on Neurotransmitter‐related Disorders (iNTD: https://www.intd‐registry.org), and the registry for mitochondrial disorders (mitoREGISTRY and mitoGLOBAL: https://www.mitonet.org) were developed [24, 25]. These registries enabled secure remote data collection and centralized storage of pseudonymized data in the context of an observational study adapted to the local legal context of the participating healthcare providers (HCPs) [14].

### 2.1 Evolution of Data Models for Patient Registries for Inherited Metabolic Disorders

Patient registries that collect data in a structured way are an effective tool for addressing the above aspects under real‐world conditions. However, as a recent systematic review showed, many patient registries for rare diseases are hampered by quality issues, particularly with regard to the use of a common data set and an ontological coding language [19]. Furthermore, many registries still do not fully comply with the European Rare Disease Registry Infrastructure (ERDRI) requirements and do not follow the FAIR principle [18]. Improvement in these areas would clearly enhance the knowledge base, promote networking of medical professionals and stakeholders at the European and international level, and establish the basis for future clinical trials and postauthorization monitoring of orphan drugs for all IMDs [14]. To fulfil these requirements and to offer a patient registry for all known IMDs, the Unified Registry for Inherited Metabolic Disorders (U‐IMD) was established in 2019 under the umbrella of the European Reference Network for Hereditary Metabolic Disorders (MetabERN) [15]. The registry implements the complete set of common data elements (CDE) which were suggested by an EU expert group as essential components for all rare disease registries (EU‐RD‐platform) [26]. By using systematic and controlled vocabularies for coding diagnosis and describing phenotypic abnormalities (i.e., nosology of the IEMbase, terms of the Human Phenotype Ontology [HPO, https://hpo.jax.org/], the coding system of the Human Metabolome Database [HMDB, https://www.hmdb.ca], etc.) as well as the usage of core data sets, the U‐IMD registry enables a high level of semantic interoperability between different platforms [15]. The term “semantic interoperability” refers to a concept that is primarily used in the fields of information systems, data exchange, and computer science. It means that different systems not only exchange data but also understand the meaning of the data in the same way.

**FIGURE 1:**
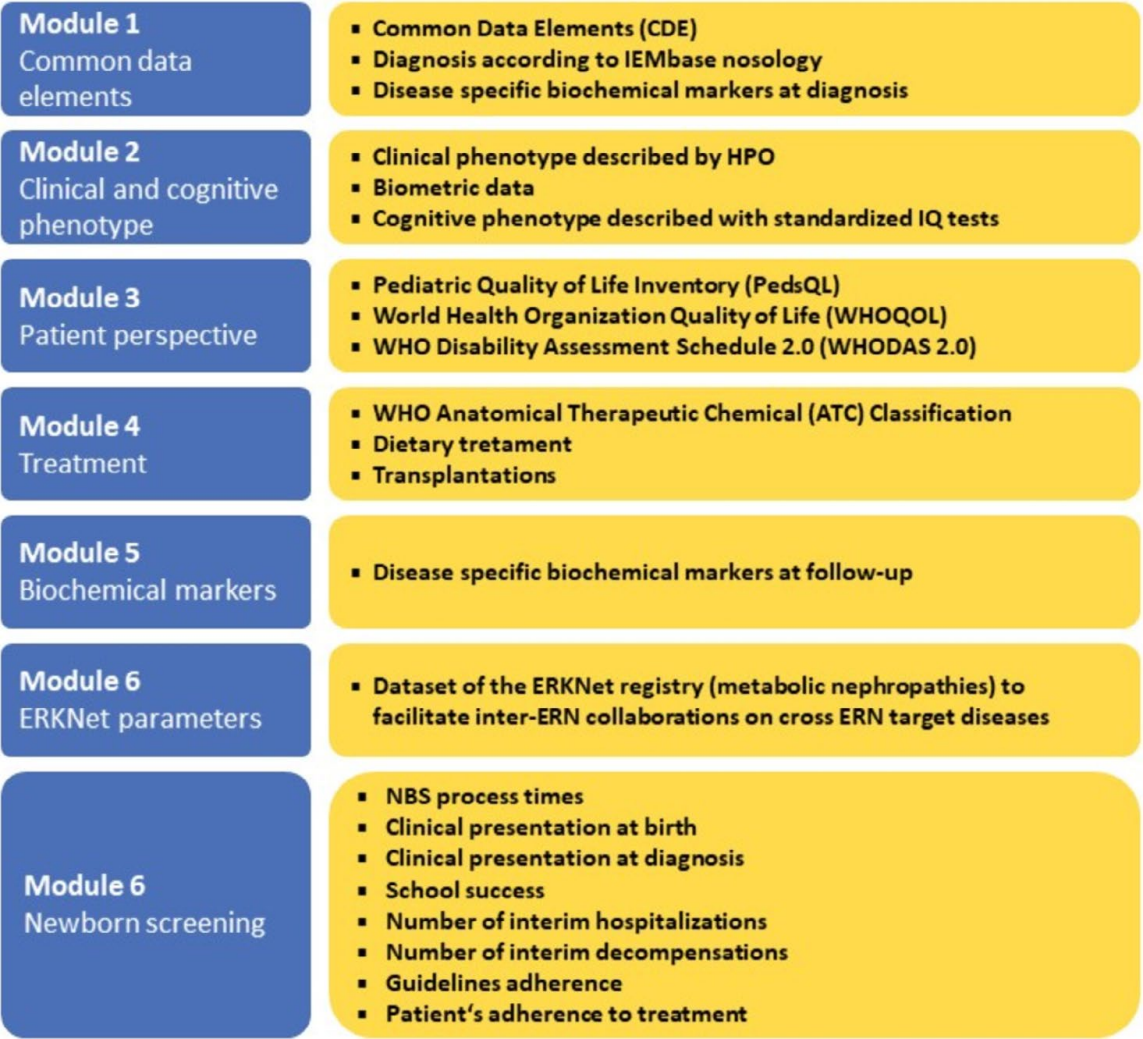
Overview of the modular structure of the U‐IMD registry, highlighting the contents of the newly added module for newborn screening.

By April 2025, more than 3800 individuals with a confirmed IMD from 37 HCPs in 14 different European and International countries have been registered. The U‐IMD registry is designed in a modular fashion, allowing phenotypic characterization of patients on different levels according to data availability, underlying disease, or research question and offering flexibility over time by adding additional modules to address new data sources, focus diseases, and facilitate new collaborations. This is exemplified by the recent addition of a module focused on newborn screening and research questions related to long‐term outcomes as a consequence of differing diagnostic journeys (Figure 1). The NBS module was developed as a collaborative effort under the umbrella of the Screen4Rare ERN Expert Platform for Newborn Screening (https://screen4rare.org/ern‐expert‐platform/) and is used for the evaluation of NBS programmes across various screened diseases and EU countries within the framework of the European Rare Diseases Research Alliance (ERDERA, https://erdera.org/).

## 3 Disease Course in Real‐World Settings: Natural History Studies

A practical example of the use of structured, real‐world, long‐term registry data is the development of natural history studies, as registry‐based observational studies can reveal phenotypic diversity and long‐term disease progression [25, 27, 28]. For instance, these studies identified an increased risk of prematurity, small‐for‐gestational‐age births, and congenital microcephaly in individuals with neurotransmitter‐related disorders [28]. Furthermore, strategies to distinguish severe and attenuated disease courses were established, resulting in a more reliable case definition, as well as improved indication to treat and therapeutic stratification, such as in isovaleric aciduria [29] and cystathionine β‐synthase (CBS) deficiency [30, 31]. Following individuals with an IMD into adulthood resulted in the first description of so far unknown disease manifestations, such as prolactinomas in inherited disorders of biogenic amine metabolism [32], the manifestation of multiorgan involvement in individuals with methylmalonic and propionic acidaemia [33], and the impact of the biochemical subgroup on cognitive function, white matter abnormalities, and chronic kidney disease in glutaric aciduria type 1 [34, 35].

In the context of trial readiness, a synthetic real‐world evidence (RWE) group from natural history studies can serve as a control arm in clinical trials (Single‐arm clinical trials [SAT]), particularly in rare IMDs, where traditionally designed RCTs are often infeasible due to the inability to recruit enough patients or the unwillingness of patients or trial leaders to randomly assign anyone to placebo, as in ceroid lipofuscinosis type 2 (CLN2) Batten disease [36].

However, since real‐world control groups are not equivalent to randomized controls, rigorous methodological and statistical adjustments after data collection, including post hoc matching and subgroup analysis, have to be applied to achieve credible comparative effectiveness data.

Recently, novel statistical approaches like the Bayesian statistical method have been proposed to overcome limitations in patient sizes and limited statistical power [37]. In such a Bayesian framework, a natural history study can serve as a “prior belief,” meaning it provides with existing natural history data a structured, quantitative estimate of how the disease progresses without treatment. These estimates form the baseline assumption, which can be compared with the effect in a clinical trial, for example, SPR1NT trial for SMA [38]. Bayesian frameworks also enable an adaptive trial design by constantly updating probabilities based on new data coming in, allowing real‐time decision‐making based on the current best estimate of treatment benefit [39].

## 4 PROMs and PREMs: Entering the Patient Perspective

Clinical assessments often miss subjective but meaningful parts of the natural history. Therefore, in addition to clinical assessment, patient registries containing caregiver impact or health‐related quality of life (HRQOL) questionnaires should also be used to capture the perspectives or experiences of patients with rare diseases directly (patient‐reported outcomes and experience measures, PROMs and PREMs). PROMs and PREMs enhance the understanding of the natural phenotype in rare diseases, can identify clinically meaningful endpoints, characterize the disease burden over time, record unmet medical needs and concerns, financial challenges, the quality of life of patients and caregivers, and, importantly, clarify how patients perceive trial design or research burden [40, 41, 42]. This comprehensive information contributes to a more complete picture of patients' health status and caregiver burden, and indicates unmet needs, e.g., for sufficient information about the neurodevelopment prognosis of children with UCDs [43, 44] or the impact of full organ liver transplantation on patients with organic acidurias, UCDs, and maple syrup urine disease [45]. Understanding the patient perspective also significantly contributes to the design of a new clinical study and to the definition of study endpoints that are truly relevant to patients [46].

## 5 Utilizing Known Evidence Through Meta‐Analyses and Mathematical Modelling

While disease registries allow a thoroughly structured, protocol‐driven data collection, they are at the same time highly resource‐intensive in terms of time and personnel, which might not be available for the plethora of orphan conditions. An alternative is statistical methods that combine the results of multiple independent studies on the same topic to produce a single, more powerful conclusion.

### 5.1 Meta‐Analysis and Quantitative Retrospective Natural History Modelling (QUARNAM)

Meta‐analyses aim to summarize and synthesize results from multiple independent studies (usually clinical trials or observational studies) to estimate an overall effect size or outcome. With the use of published aggregate data, recent meta‐analyses confirmed the high risks of early onset disease manifestation in all UCDs apart from female ornithine transcarbamylase deficiency (mOTC‐D) [47] and the positive impact of newborn screening on the neurological outcome in individuals with maple syrup urine disease and glutaric aciduria type 1 [21, 22].

The QUARNAM aims to reconstruct disease progression over time using historical, observational patient data from retrospective data without treatment. QUARNAM has a comparable relation to a published case study as a meta‐analysis has to an individual published (aggregated) study [23]. It applies biostatistical methods such as Kaplan–Meier estimates, cluster analyses, regression models, binary decision trees, word clouds and geographical mapping with a lower effort to define important clinical outcome measures for an ultra‐rare disease based on published case reports or case series [23]. To this end, QUARNAM has provided information on diagnostic delay, survival characteristics as well as biomarker‐phenotype‐correlations and geographical distribution pattern in an overall cohort of more than 800 individuals worldwide in several neurogenetic disorders with a known prevalence of less than 100 cases and 1 in 1 000 000 such as α‐mannosidosis [48], Farber disease [49], galactosialidosis [50], Krabbe disease [51], molybdenum cofactor deficiency [52], mucopolysaccharidosis type VII [53], and sialic acid storage disease [54]. Within a particular drug development program, the choice of method to quantitate the natural history of a rare disease may encompass a combinatory approach including QUARNAM depending on prevalence of the condition, resource availability, and estimated time to study completion.

### 5.2 Scoring Systems to Evaluate Disease Course and Early Predictive Modelling of Disease Severity

A reliable and comparable assessment of clinical signs and symptoms is one of the key requirements for (I) an objective and valid description of the disease course, (II) differentiation of respective phenotypes, and (III) evaluation of therapeutic interventions. These are also included in the requirements for clinical trial readiness. In that context, quantifying observed clinical findings as “scores” or “rating scales” offers a promising and user‐friendly approach. Interrater variability, validity, and responsiveness are relevant features to be examined in such scoring systems. One example of such a score has been successfully applied for CLN2 disease, a rare autosomal recessive, neurodegenerative lysosomal storage disease with paediatric onset. The so‐called “Hamburg score” was designed to rate motor, visual, and verbal functions as well as the incidence of cerebral seizures [55]. Each item is scored 0–3, resulting in a total summed score ranging from 0 to 12. This score was refined with detailed definitions to ensure consistent ratings in multinational, multisite clinical efficacy studies, and it was renamed the Clinical Rating Scale (CRS) for CLN2 [56]. Currently, this scale serves as the primary outcome measure in clinical trials for the ERT with cerliponase alfa for CNL2 [36, 57].

Another approach to establish a clinical scoring system has been implemented for neuronal CLN3 to meet the requirements of trials for new therapeutic interventions, including gene therapy [58, 59]. The Unified Batten Disease Rating Scale (UBDRS) was developed in 2005 and refined in 2011 to improve its validity and reliability [60]. It assesses motor, behavioral, and functional capability along with seizure characteristics. Compared to the CRS for CLN2, the UBDRS provides a more detailed and comprehensive scoring system by including interviews with parents or caregivers and describing seizure semiology rather than solely recording seizure incidence. The UBDRS is included in inclusion/exclusion criteria and in the primary outcome measures of efficacy in the ongoing gene therapy trial for CLN3 (https://clinicaltrials.gov/study/NCT03770572).

Prediction models for disease severity are complementary to CRSs and can serve as useful tools to assist for (1) the counselling of affected individuals and their families with regard to the expected disease course and long‐term outcome at a very early stage of the disease (i.e., when genetic test results are available), (2) informed decision‐making and establishment of personalized care concepts, and (3) the design of future clinical trials in terms of severity‐adjusted group assignment to enable a reliable interpretation of the therapeutic effects of novel treatment approaches. Different methods have been used to establish a prediction model depending on disease characteristics such as age of onset, type and progression of the disease course, presence of biomarkers, availability of functional assays, and information on genotype.

**FIGURE 2:**
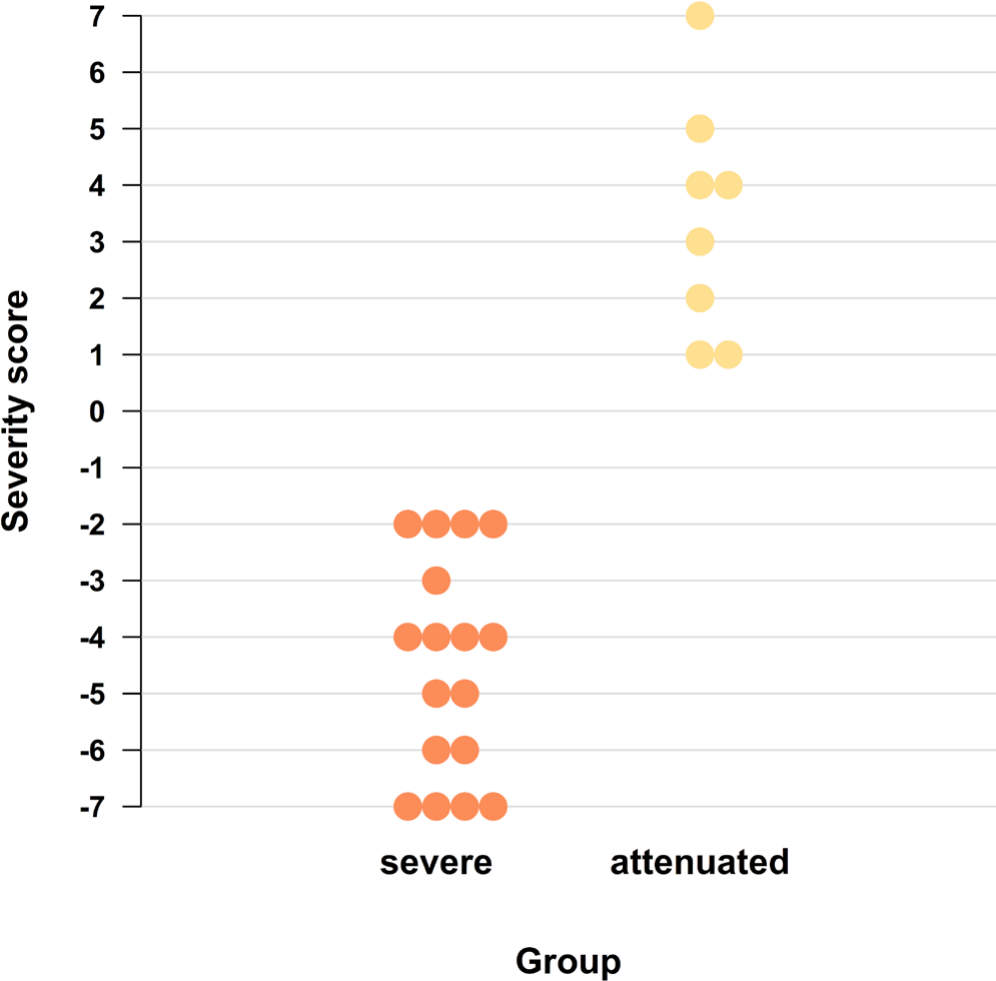
Classification of disease severity in NKH in attenuated and severe course using a scoring system with five parameters (age at onset, CSF glycine, CSF/plasma, glycine ratio, presence of corpus callosum abnormalities). Every dot represents the total score per patient. Wilcoxon–Mann–Whitney test, *p* ≤ 0.0001. Score range = −7 to +7. *Source:* Adapted from [63].

Nonketotic hyperglycinemia (NKH) is a neurometabolic disorder with a severe and attenuated disease course, presenting with neonatal‐onset epileptic encephalopathy and high lethality versus choreatic movement and psychiatric disorders, respectively [61, 62]. Therapeutic options for the severe form are highly limited. A multiparametric severity prediction model published in 2022 evaluates disease‐relevant and easily accessible medical information regarding its sensitivity and specificity for each disease course [63]. It combines clinical parameters (age at onset), biochemical markers (cerebral spinal fluid [CSF] glycine concentration and plasma/CSF glycine ratio), radiological findings (disease‐specific magnet resonance imaging [MRI] pattern), and genetic data (in silico prediction of causative genetic variants, Figure 2).

Quantification of the protein dysfunction pathomechanistically underlying a genetic (neurometabolic) disease and its correlation with real‐world biochemical and clinical disease characteristics systematically collected in structured natural history studies is another powerful strategy that proved successful for prediction modelling for the three most prevalent UCDs, that is, mOTC‐D, citrullinemia type 1 (CTLN1), and argininosuccinic aciduria (ASA) [64, 65, 66]. Residual enzymatic activities, as determined by a (biallelic) mammalian expression system, not only predicted the severity of the initial disease manifestation and the number of subsequent metabolic decompensations during the disease course, as well as the neurocognitive long‐term outcome, but also enabled reliable differentiation between individuals with a severe or attenuated phenotype based on the underlying pathogenic variant(s) [67]. Importantly, the prediction model was already successfully applied for the severity‐adjusted and comparative evaluation of currently available key diagnostic (i.e., newborn screening for CTLN1 and ASA [68]) and therapeutic interventions (i.e., liver transplantation [7], initial dialysis [69], adding significantly to the knowledge in the field of UCDs). The modelling approach might serve as a blueprint for further intoxication‐type metabolic diseases for the prediction of individual disease severity, risk stratification, and clinical trial readiness.

### 5.3 Personalized Metabolic Whole‐Body Models

Artificial intelligence (AI) and machine learning (ML) are widely used and can support diagnosis and medical decision‐making in a variety of ways. In newborn screening, for example, ML has been shown to improve specificity when used as a “digital tier” [70, 71]. Extending the consideration from individual metabolic profiles to the entire metabolism, mathematical models of complex metabolic networks that integrate the genotype and epigenetic influences and predict the clinical phenotype as possible “digital twins” are visions that have already been modelled as whole‐body models (WBM) for male and female adults [72] and recently for newborns and infants (infant WBM), which represent the rapidly growing metabolism of the first 180 days of life [73]. Each WBM contains over 80 000 biochemical reactions in an anatomically and physiologically consistent manner and can be parameterized with physiological, nutritional, and metabolic data. Importantly, these WBMs can also be personalized using clinical and omics data, for example, metabolomic and metagenomic data [72]. Further, these models offer the possibility of being linked to pharmacological data that not only predict the course of disease but also the therapeutic effects of nutrition and medication, for example in IMDs [74].

## 6 Best‐Practice: Consensus Guidelines

In the same way that structured data collection in patient registries is an essential prerequisite for conducting natural history studies, the use of meta‐analyses and QUARNAMs and their evaluation of the available evidence is an essential requirement for developing clinical, consensus‐based guidelines. Clinical guidelines are at the same time essential tools for clinical trial preparation and conduction, since they describe diagnostic criteria and phenotypic classifications, define standards of care (SoC) and validated clinical endpoints, address gaps in clinical care and suggest reliable follow‐up parameters including timing for assessments [14].

Nowadays, there are various clinical guidelines for IMDs, most of which have been compiled by international consortia [75, 76, 77, 78]. Although their availability and quality can vary greatly depending on the disease, they nevertheless contribute significantly to standardizing clinical, diagnostic, and therapeutic procedures at the international level. Recently, careful biochemical and clinical evaluation of more than 300 individuals with confirmed CBS deficiency resulted in the development of comprehensive criteria for the classification of pyridoxine responsiveness [30]. At the same time, with the help of the structured registry data, the various therapeutic procedures mentioned in clinical guidelines, for example, full organ liver transplantation and hemodialysis for urea cycle defects, could be evaluated in terms of their efficiency and long‐term prognosis [7, 69]. While liver transplantation prevented acute life‐threatening metabolic decompensations without further need of protein restriction or nitrogen‐scavenging therapy, it was not associated with improved neurocognitive outcomes in the investigated UCDs [69].

## 7 Safety First: Quality‐Assured Follow‐Up and Postauthorization Studies

Postauthorization safety studies (PASS) are systematically planned, noninterventional or interventional studies conducted after a drug has been approved. They are an integral part of pharmacovigilance and serve to continuously evaluate the safety and benefit profile of a drug under everyday conditions. They play an important role in the field of IMDs and new (gene) therapies, which are often approved after a relatively short study phase with a small number of patients. For example, for the recently approved gene therapy of AADC deficiency (Upstaza), this means that there are considerable uncertainties regarding its long‐term effect on the health of the patient, the cost effectiveness, and the related side effect profile. In order to responsibly address these uncertainties, the cross‐border networks, with the help of the experience gained from the scientific patient registries, developed proposals for the collection of reliable follow‐up data in quality‐assured follow‐up care [79]. These proposals, together with pivotal parameters, can be modularly integrated into existing scientific disease registries, which often already collect this type of longitudinal data. Subsequent collaboration between marketing authorization holders (MAH) and the scientific consortia and their patient registries in a public–private partnership would be useful to achieve the objectives of certain drug safety measures while reducing fragmentation and duplicate data storage [80]. Successful examples of this are the PASS studies recently conducted by E‐HOD and EIMD for the orphan drugs Cystadane (active substance: betaine anhydrous; [80]) and Ravicti (active substance: glycerol phenylbutyrate; EUPAS no. 17267). In both cases, the PASS studies were conducted according to a protocol designed by the MAH together with the scientific registries and approved by the EMA's Pharmacovigilance Risk Assessment Committee (PRAC).

## 8 Conclusion and Outlook

**FIGURE 3:**
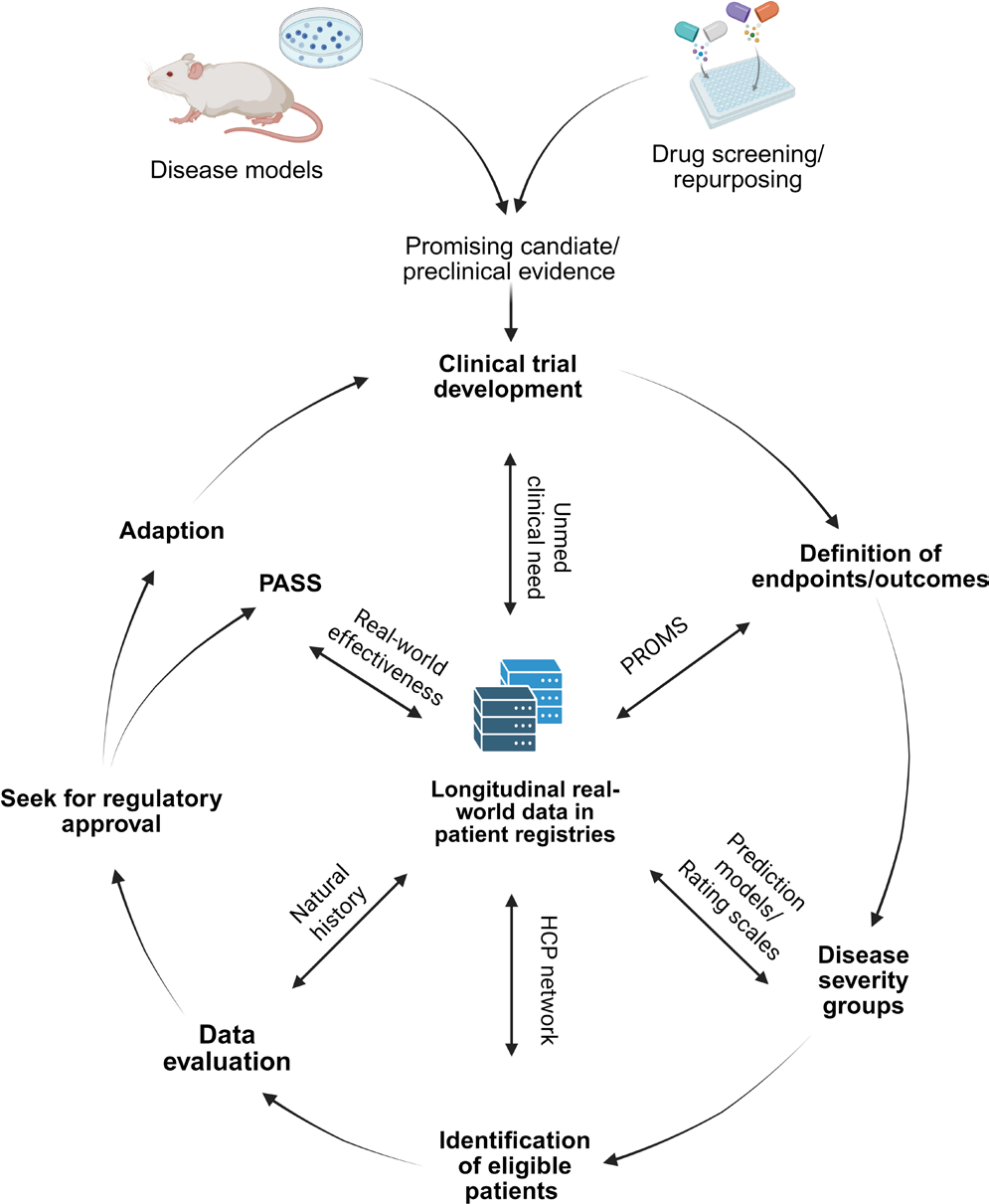
Iterative cycle emphasizing the importance of comprehensive collection of longitudinal real‐world data in patient registries as a basis for understanding the natural history of IMDs and its importance for trial readiness.

Due to the unique challenges posed by small, heterogeneous patient populations and limited historical data, the preparation for and execution of clinical trials for innovative treatments in IMDs must be adaptive, flexible, and highly collaborative with patient groups, scientific networks, and specialized health care providers. Hereby, the collection of longitudinal real‐world data in patient registries in an international collaborative framework is the cornerstone of the comprehensive understanding of the natural history (Figure 3). A multistakeholder governance model is generally considered best practice for such rare disease registries. Ownership and hosting should ideally be by neutral, sustainable entities (academic/nonprofit) with strong governance that includes patient representation.

The understanding of the natural history goes far beyond simply understanding the progression of a disease. It is a multilayered process, where the data obtained from longitudinal observational studies provide practical information on case definition, phenotype, and severity of the disease, but also contribute to patient‐relevant endpoints through PROMS. The data also feeds into guidelines, predictive models, and the development of PASS, the outcomes of which ideally contribute to an iterative cycle to further optimization of clinical trial readiness.

## Author Contributions

Conception and design of the article by Thomas Opladen and Stefan Kölker. Thomas Opladen drafted the manuscript and coordinated communications with all authors. All authors wrote parts of the manuscript, revised, and approved the final manuscript.

## Ethics Statement

The authors have nothing to report.

## Consent

The authors have nothing to report.

## Conflicts of Interest

The authors declare no conflicts of interest.

## Data Availability

Data sharing is not applicable to this article as no datasets were generated or analysed during the current study.
